# Ultrasound combined with FeSO_4_ facilitated the occurrence of ferroptosis in *Vibrio parahaemolyticus*

**DOI:** 10.1016/j.ultsonch.2024.107080

**Published:** 2024-09-23

**Authors:** Shurui Peng, Lishan Yao, Xiaolin Zhu, Wei Ge, Jiakun Deng, Hongbo Li, Dan Xu, Liangbin Hu, Haizhen Mo

**Affiliations:** School of Food Science and Engineering, Shaanxi University of Science and Technology, Xi’an 710021, China

**Keywords:** Ultrasound, *Vibrio parahaemolyticus*, FeSO_4_, Synergistic sterilization, Ferroptosis

## Abstract

Ultrasound (US) as a sustainable non-thermal sterilization technology that is employed either independently alone or in combination with other processing methods to eliminate food-borne pathogens in the food industry. In the present study, the synergistic effects of US combined with FeSO_4_ against *Vibrio parahaemolyticus* were investigated. The results demonstrated that the combination of ultrasound and FeSO_4_ had an excellent bactericidal activity on *V. parahaemolyticus*. Treatment with US (100 W) and FeSO_4_ (8 μM) for 15 min could kill more than 99.9 % cells. Furthermore, the observed cell death was identified as classical ferroptosis, characterized by ferroptosis hallmarks including iron-dependent, ROS burst, membrane damage and lipid peroxide accumulation. Addition of ferroptosis inhibitor liproxstatin-1 alleviated the cell death induced by the combination treatment. Transcriptome analysis further revealed that the US-FeSO_4_ treatment significantly influenced pathways related to fatty acid metabolism, ferroptosis, biofilm formation, RNA degradation, oxidative phosphorylation and other key processes, which likely contributed to the occurrence of ferroptosis. Based on these findings, we speculated that cavitation effect of US promoted the entry of Fe^2+^, leading to the generation of free radicals primarily responsible for ferroptosis by US-FeSO_4_. Taken together, this study provides valuable insights into the biological pathway involved in ultrasound sterilization and presents an alternative strategy to eradicate microorganism in food products.

## Highlights

• Ultrasound (US) combined with FeSO_4_ had a synergistic bactericidal effect.
• US+FeSO_4_ facilitated the occurrence of ferroptosis in *V. parahaemolyticus*.
• The cavitation effect of US promoted the entry of Fe^2+^.

## Graphical abstract

**Figure:**
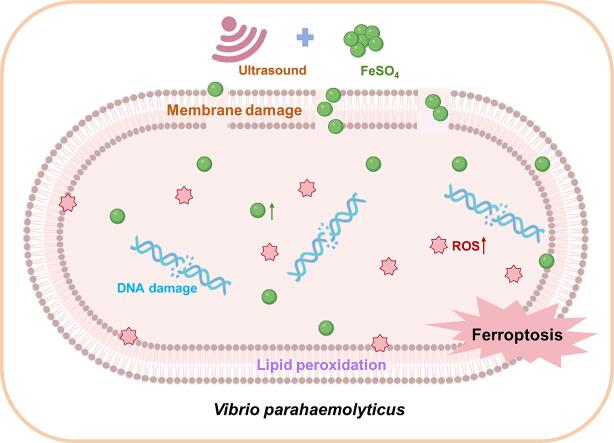

## 1 Introduction

*Vibrio parahaemolyticus* is a prevalent food-borne pathogen that widely distributes in marine environments and frequently contaminates seafood [1], [2]. Consumption of contaminated water and seafood can cause vomiting, gastroenteritis and diarrhea in humans, which has aroused global public attention [3], [4], [5]. Antibiotics are the most common and effective strategies for treating *V. parahaemolyticus* infection, while the development of drug resistance and multidrug-resistant strains pose a massive challenge for its control [6], [7]. Consequently, there is a critical need for novel antimicrobial strategies to mitigate *V. parahaemolyticus* contamination.

Ferroptosis is a novel and unique cell death modality driven by highly iron-dependent lipid peroxidation [8]. It has attracted extensive attention in recent years owing to its tremendous potential in the treatment of various diseases [9], [10], [11]. Ferroptosis can be triggered via the extrinsic pathways involving drugs, small-molecule compounds, hypoxia, and radiation [12]. Our previous study has shown that ferrous sulfate could efficiently kill *V. parahaemolyticus* through a ferroptosis-like mode, highlighting the pivotal of Fe^2+^ influx in this process [13]. Inspired by the discovery, it is supposed that whether promoting the entry of Fe^2+^ could further induce ferroptosis in *V. parahaemolyticus*.

Ultrasound as a novel and green non-thermal physical sterilization technology has gained a great deal of interest in food industry [14], [15]. The antimicrobial efficacy of ultrasound on microorganisms primarily stems from acoustic cavitation, wherein ultrasound waves can induce the formation and rapid collapse of cavitation bubbles in the liquid medium [16]. This phenomenon generates intense liquid shear forces and shock waves, exerting pressure on bacterial cell membrane [17], [18]. Additionally, the resultant high pressure and temperature enhance mass transfer rates and promote the generation of free radicals, leading to the disruption of the cell [19]. Building upon this principle, we speculated whether the physical or chemical effects of ultrasound could facilitate the entry of Fe^2+^ into cells, potentially triggering the occurrence of ferroptosis. Therefore, this study aimed to evaluate the bactericidal effects of ultrasound combined with FeSO_4_ against *V. parahaemolyticus* and explore its underlying mechanism. These findings would not only provide an alternative strategy for enhancing food safety, but also contribute to expanding our understanding of the diverse biological pathways influenced by ultrasound.

## 2 Materials and methods

### 2.1 Bacterial strains and regents

*V. parahaemolyticus* RIMD 2210633 used in this study was derived from Chinese Center for Disease control and Prevention (CCDCP). Bacteria cells were incubated in lysogeny broth (LB) medium with 2 % NaCl at 37 °C with shaking at 200 rpm. *Staphylococcus aureus* ATCC 6538 was purchased from China General Microbiological Culture collection Center (CGMCC). Cells (approximately 10^8^ CFU/mL) were collected by centrifugation at 8000 × g for 5 min and resuspended in 0.9 % NaCl for subsequent use. Ferrous sulfate (FeSO_4_) and other chemical agents were analytical grade.

### 2.2 Ultrasonic treatments

The synergistic effect of US and FeSO_4_ was evaluated by drop plate method [20]. The bacterial suspension was exposed to different concentrations of FeSO_4_ (0, 2, 4, 8, 16 μM), followed sonicated under 60 W, 80 W, 100 W, 120 W for 10 min, 15 min, 20 min. After treatment, bacteria suspension was centrifuged at 8000 × g for 5 min and washed with 0.9 % NaCl. Cells were diluted in a 10-fold gradient with 0.9 % NaCl, 5 μL of dilutions were dropped onto LB agar. The survival rates were calculated by counting the cell population after incubated at 37 °C for 12 h.

### 2.3 Cell viability analysis

The cell viability was further analyzed by employing fluorescent probe Propidium iodide (PI, ST511, Beyotime Institute of Biotechnology, China) [13]. Briefly, cells exposed to 8 μM FeSO_4_ were sonicated under 100 W for 15 min. After centrifugation, the pellets were resuspended with 0.9 % NaCl and loaded with 1 μg/mL PI (ST511, Beyotime Institute of Biotechnology, China) at 37 °C for 30 min. PI-strained cells were evaluated by flow cytometer (NovoCyte 3000, Agilent, USA) and inverted fluorescent microscope (IX73P1F, Olympus, Japan).

### 2.4 Scanning electron microscopy (SEM) observation

SEM was performed referring to the method of Zhou with slight modifications [21]. After treated with FeSO_4_ and US, the samples were washed with PBS and fixed with 2.5 % glutaraldehyde at 4°C overnight. Then, the cells were washed with PBS for three times and dehydrated with alcohol at a gradient concentration (from 30 % to 100 %) for 10 min. After further resuspension with isoamyl acetate, the cells were coated with gold under vacuum and observed under a scanning electron microscope at a magnification of 20,000×.

### 2.5 Determination of intracellular ROS

The endogenous ROS generation was detected using 2′,7′-dichlorofluorescin diacetate (DCFH-DA) (HY-D0940, MCE, USA) according to the manufacturer’s instructions. The DCFH-DA probe (final concentration at 10 μM) was loaded into bacterial suspension. After incubation at 37 °C for 30 min in the darkness, samples were collected and treated with FeSO_4_ and US. The intracellular levels of ROS were assayed by flow cytometry and inverted fluorescence microscope.

### 2.6 Determination of intracellular Fe^2+^

The specific fluorescent probe FeRhoNox-1 (GC901, Goryo Chemical, Japan) was employed to measure the content of intracellular Fe^2+^ with reference to previous study [22]. Cells exposed to FeSO_4_ and US were incubated with 5 μM FeRhoNox-1 probe and incubated at 4 °C for 1 h in the darkness. Cells were washed with 0.9 % NaCl and detected by flow cytometry and inverted fluorescence microscope.

### 2.7 Lipid peroxidation assay

The levels of lipid peroxidation in *V. parahaemolyticus* cells were monitored by using C11-BODIPY (D3861, Thermo Fisher Scientific, USA) probe [23]. Cells with different treatments (FeSO_4_ and US) were incubated with 1 μM C11-BODIPY at 37 °C for 30 min in the darkness. Subsequently, lipid peroxidation levels were detected by flow cytometry and inverted fluorescence microscope.

### 2.8 Transcriptomic analysis

Samples were collected of cells subjected to ultrasound at 100 W for 15 min after being exposed to 8 μM FeSO_4_, while cells without any treatment were set as the control group. All samples were washed with 0.9 % NaCl and quickly frozen in liquid nitrogen. Total RNA extraction was performed using TRIzol® Reagent (Invitrogen, Carlsbad, CA, USA) according to the manufacturer's instructions. RNA sequencing and data collection were performed by BGI Genomics (Guangdong, China). The bioinformatics analysis of RNA-Seq data was referring to Mo [24]. The DEGs (differentially expressed genes) were identified by using DESeq2 with *p*-value < 0.05 and a fold change > 2 or fold change < 0.5. GO (Gene Ontology) functional enrichment and KEGG (Kyoto Encyclopedia of Genes and Genomes) pathway enrichment were performed based on DEGs. A *p*-value less than 0.05 indicated statistically significant enrichment and all data were the average of three replications.

### 2.9 Application of combined treatment on salmon

#### 2.9.1 Preparation of salmon sample

Fresh salmon was purchased from local supermarket (Freshippo) and was prepared according to the method of Zhao et al. [13] with slight modifications. The salmon samples were cut into 2 cm × 2 cm × 0.5 cm pieces and immersed in *V. parahaemolyticus* suspension (10^7^ CFU/mL) for 30 min. The treatments included US (100 W), FeSO_4_ (16, 32 μM) and US+FeSO_4_. After treatment, samples were homogenized in a sterile homogenization bag containing 10 mL NaCl for 3 min. The mixtures were serially diluted with NaCl and dropped onto LB agar (2 % NaCl). The survival rates were calculated by counting the cell population after incubated at 37 °C for 12 h.

#### 2.9.2 Physical and chemical indicators of salmon

To evaluate the effect of different treatments on the characteristics of salmon, the color measurement, texture profile analysis, shearing force measurement, and sensory analysis were conducted.

Color changes in salmon were measured with a Spectrophotometer (CM-5, Konica Minolta). Five readings were recorded at different surface points. Color was described using the CIELAB scale: lightness (L*), green to red hue (a*), and blue to yellow hue (b*).

Salmon samples were detected with a texture analysis (TPA; TA.XT PlusC, Britain) with a P/75 probe. The pre-test, test and post-test speeds were set at 1.00, 1.00 and 10.00 mm/s, respectively. Each test for the different treatments was performed at a uniform position.

The sensory evaluation was evaluated following the requirements of the Chinese standard GB/T 22210–2008. Ten trained accessors (Male = 5; Female = 5; mean age = 26) were recruited among postgraduate students of School of Food Science and Engineering, Shaanxi University of Science and Technology (Xian, Shaanxi). The sensory evaluation index included appearance (glossiness), odour (freshness), and flavor (taste). The sensory criteria were as follows: 5 = very good; 3 = medium; 1 = poor.

### 2.10 Statistical analysis

All experiments were performed in three independent biological replicates. All graphs were generated using GraphPad Prism® version 9 and FlowJo v10.8.1. The data were displayed as mean ± standard deviation (SD). Statistically significant differences were analyzed using one-way analysis of variance (ANOVA) by IBM SPSS Statistics 20. Differences of *p* < 0.05 were considered statistically significant.

## 3 Results

### 3.1 Ultrasound combined with FeSO_4_ efficiently killed *V. parahaemolyticus*

**Fig. 1:**
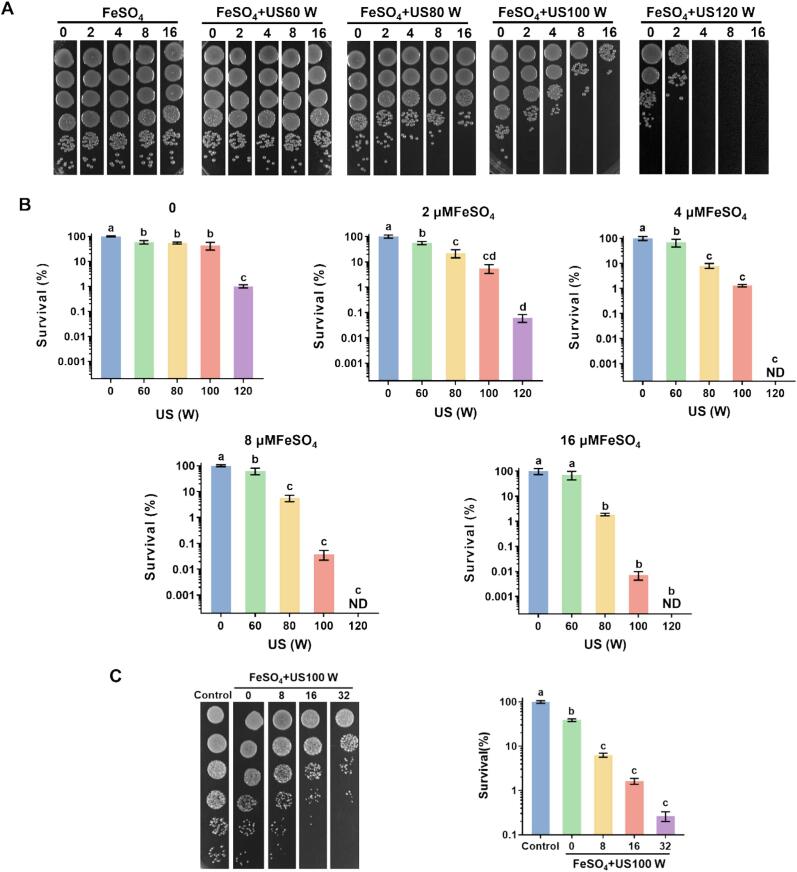
Bactericiadal effects of the combination of ultrasound and FeSO_4_ against *V. parahaemolyticus*. (A) Colony formation serially-dilutional cells exposed to different combined treatments. (B) The survival of *V. parahaemolyticus* cells exposed to different combined treatments. ND indicated no colony formation. (C) Colony formation and survival of *Staphylococcus aureus* exposed to different combined treatments. Different letters above the bars indicated that the mean values of three replicates were significantly different among different treatments (*p* < 0.05).

The bactericidal effects of *V. parahaemolyticus* by independent or combined treatments of FeSO_4_ and US were shown in Fig. 1. The treatments with FeSO_4_ alone at various concentrations (2, 4, 8, 16 μM) for 15 min showed little effect on the viability of *V. parahaemolyticus*. For individually treatment of different ultrasonic powers for 15 min, the bacterial population had no significant decrease among US treatments with 60–100 W. While the number of viable bacteria obviously reduced after being treated with 120 W US, almost 99 % of *V. parahaemolyticus* were dead (Fig. 1B). Notably, the combined treatments of FeSO_4_ and US had significant reductions in bacterial population compared with US and FeSO_4_ independent treatment, exhibiting a power and dose-dependent manner. These results indicated FeSO_4_ and US had a synergetic bactericidal effect on *V. parahaemolyticus*. Treatment with US (100 W) + FeSO_4_ (8, 16 μM), US (120 W) + FeSO_4_ (2 μM) for 15 min, more than 99.96 % cells were killed, and no growth of *V. parahaemolyticus* was detected at US (120 W) + FeSO_4_ (4, 8, 16 μM) (Fig. 1A, B). Therefore, the combination of US (100 W) and FeSO_4_ (8 μM) was determined to be the optimal bactericidal condition against *V. parahaemolyticus*, which was selected for further investigation in this study. In addition, the combination of US (100 W) and FeSO_4_ also exhibited a significant bactericidal activity to Gram-positive bacteria *Staphylococcus aureus*. Taken together, our results indicated that the combination of US and FeSO_4_ had excellent bactericidal activity on both Gram-positive and Gram-negative bacteria.

### 3.2 Ultrasound combined with FeSO_4_ facilitated the morphological changes of *V. parahaemolyticus*

**Fig. 2:**
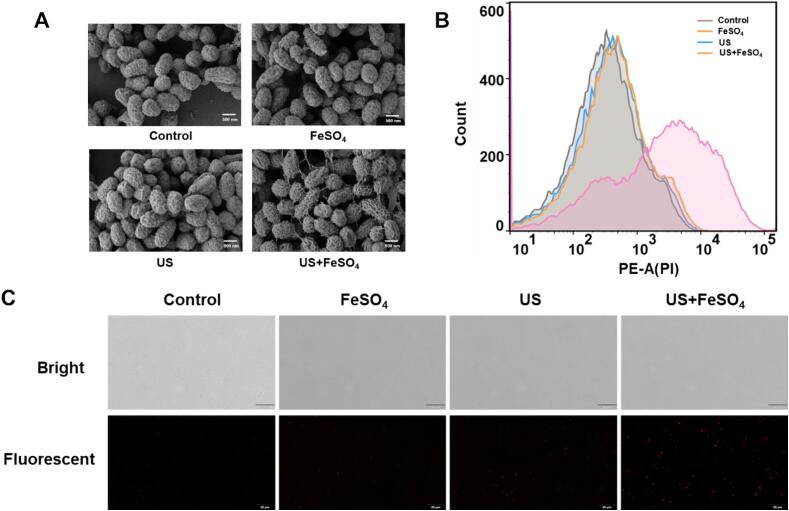
Effect of the combination of ultrasound and FeSO_4_ on the morphology changes and survival of *V. parahaemolyticus*. (A) The morphology of *V. parahaemolyticus* observed by SEM after combined treatments. (B) Histograms of PI- staining cells exposed to ultrasound and FeSO_4_ through flow cytometry. (C) Images of PI- staining cells under fluorescence microscope after exposed to ultrasound and FeSO_4_.

The morphological changes of *V. parahaemolyticus* were further monitored after being treated with US and FeSO_4_ through SEM. As shown in Fig. 2A, cells in control group exhibited short rod-like morphology covered with intact and full surfaces. After independent treatment of US and FeSO_4_, majority of cells exhibited no apparent differences compared with the control group. In contrast, the microscopic morphology of *V. parahaemolyticus* treated with US+FeSO_4_ were collapsed and shrinkage, along with observable adhesions among the cells.

As a nuclear staining reagent, PI can only traverse damaged cell membranes and bind with nucleic acid, leading to a positive staining [25]. In the present study, PI straining was utilized to further detect the cell membrane integrity and confirm the mortality of *V. parahaemolyticus*. As shown by flow cytometry, no positive straining was observed in the US and FeSO_4_ treatments alone (Fig. 2B). While in the combined treatment, the fluorescent intensity of PI straining was significantly increased, shifting towards longer wavelengths. In addition, PI-positive cells were observed by florescent microscopy (Fig. 2C). These findings above indicated that the combined treatment of US and FeSO_4_ irreversibly damaged the cell membrane, which is a crucial factor in their bactericidal effect against *V. parahaemolyticus*.

### 3.3 Ultrasound combined with FeSO_4_ triggered ROS eruption

**Fig. 3:**
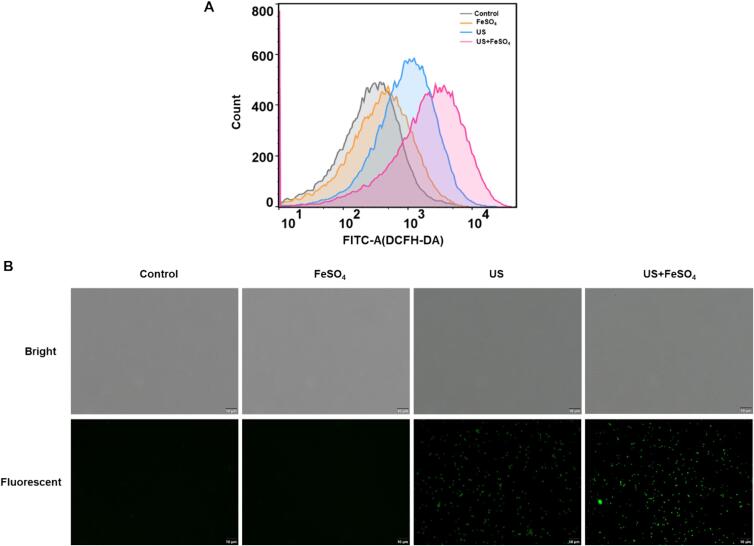
Changes of intracellular ROS in *V. parahaemolyticus* after ultrasound and FeSO_4_ treatments. Intracellular ROS was evaluated with DCFH-DA as a probe through flow cytometry (A) and fluorescent microscopy (B).

Excessive ROS production causes oxidative stress in cells, resulting in cell membrane damage and cell death [26]. Consideration the possible role of ROS in the bactericidal activity of US+FeSO_4_ on *V. parahaemolyticus*, DCFH-DA was employed to determine the level of intracellular ROS. Results showed that the florescence peaks of all treated group shifted towards longer wavelengths compared with the control group (Fig. 3A). Remarkably, the fluorescence intensity in US+FeSO_4_ group was stronger than those in the US and FeSO_4_ treatments alone, indicating synergetic treatments of US and FeSO_4_ triggered more ROS burst in *V. parahaemolyticus* (Fig. 3B).

### 3.4 Ultrasound combined with FeSO_4_ promoted the entry of Fe^2+^

**Fig. 4:**
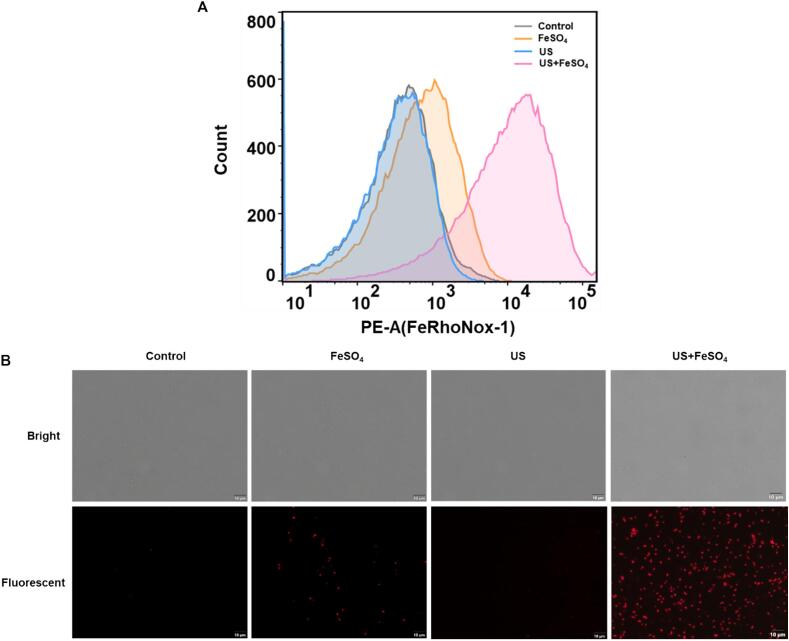
Changes of intracellular Fe^2+^ levels in *V. parahaemolyticus* after ultrasound and FeSO_4_ treatments. Intracellular Fe^2+^ levels were evaluated with FeRhoNox-1 as a probe through flow cytometry (A) and fluorescent microscopy (B).

Ferroptosis is a form of programmed cell death characterized by iron-dependent lipid peroxidation [27]. To investigate whether the combination of US and FeSO_4_ facilitated ferroptosis occurrence, a potent fluorescent probe (FeRhonox-1) was used to detect the intracellular Fe^2+^ of *V. parahaemolyticus* cells. As shown in Fig. 4A, there was no significant changes after US treatment. The addition of exogenous FeSO_4_ treatment alone led to a slight increase of Fe^2+^ inside the cell compared with the control group. While US and FeSO_4_ together induced a rapid burst of intracellular Fe^2+^. Further observation under florescent microscopy were shown in Fig. 4B, which were in line with flow cytometry analysis above. Taken together, our data suggested that synergetic treatments of US and FeSO_4_ induced a superior iron-dependent cell death.

### 3.5 Ultrasound combined with FeSO_4_ led to the occurrence of lipid peroxidation

**Fig. 5:**
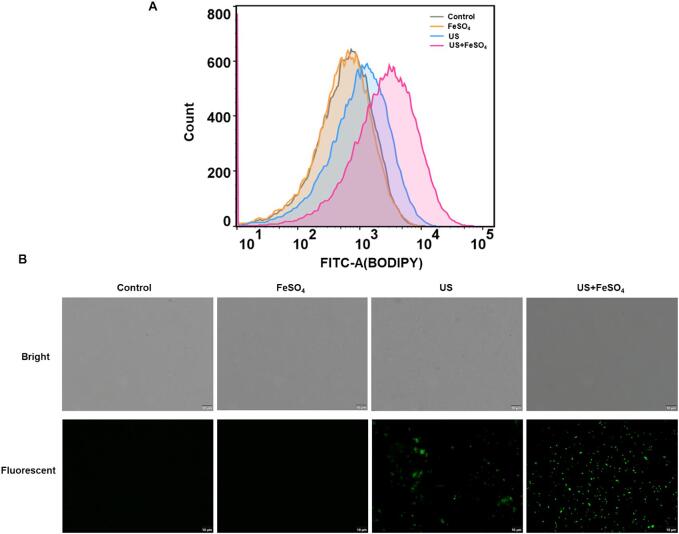
Changes of lipid peroxidation levels in *V. parahaemolyticus* after ultrasound and FeSO_4_ treatments. The level of lipid peroxidation was evaluated with C11-Bodipy as a probe through flow cytometry (A) and fluorescent microscopy (B).

Lipid peroxidation has been considered as another hallmark of ferroptosis [27]. Therefore, C11-BODIPY probe was introduced into treated cells to evaluate the lipid peroxidation level. Results revealed that the generation of lipid peroxidation in the US and FeSO_4_ treatment alone were not obviously distinguished from the control group (Fig. 5A). A significant positive shift of cell populations was observed through flow cytometry, and most cells showed strong green fluorescence under fluorescence microscopy, confirming that US+FeSO_4_ treatments led to the occurrence of lipid peroxidation (Fig. 5B).

### 3.6 Effect of ferroptosis inhibitors Liproxstatin-1

**Fig. 6:**
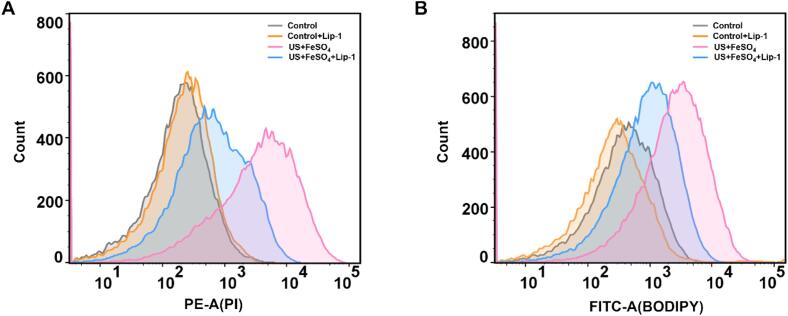
Effect of the addition of ferroptosis inhibitors Lip-1 on the cell death and lipid peroxidation in *V. parahaemolyticus*. (A) Analysis of the effects of ferroptosis inhibitors Lip-1 on the cell death induced by the combination of ultrasound and FeSO_4_. (B) Analysis of the effects of ferroptosis inhibitors Lip-1 on the level of lipid peroxidation with C11-bodipy probe.

Liproxstatin-1(Lip-1) as a specific inhibitor of ferroptosis [28], is usually used to further verify the occurrence of ferroptosis. As we expected, the addition of Lip-1 obviously rescued cell death induced by synergetic treatments of US and FeSO_4_ (Fig. 6A). Likewise, lipid peroxidation triggered by US+FeSO_4_ was also alleviated with the addition of Lip-1 (Fig. 6B). Taken together, these results based on the characteristic phenotypes including iron-dependent, ROS eruption, lipid peroxidation occurrence and Lip-1 inhibition, led us to conclude that combined treatments of US and FeSO_4_ facilitate the occurrence of ferroptosis.

### 3.7 Transcriptome analysis

**Fig. 7:**
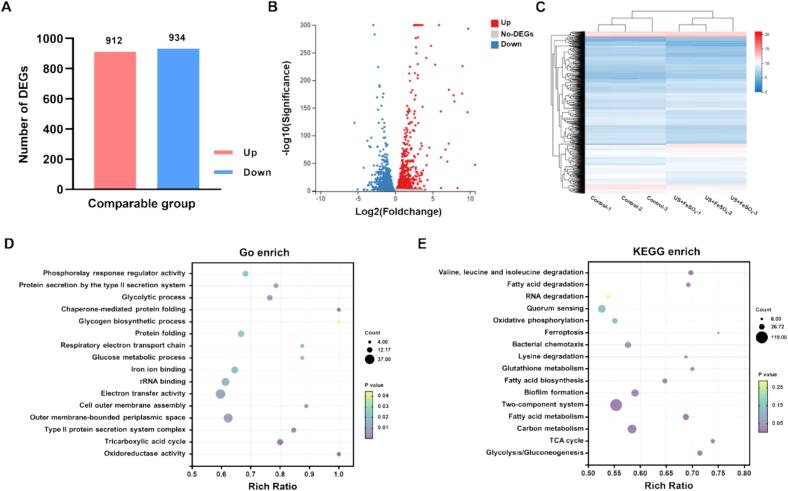
Transcriptome analysis of *V. parahaemolyticus* cells after combined ultrasound with FeSO_4_ treatments. (A) Differentially expressed genes (DEGs), (B) Volcano plot of differential gene expression, (C) Differential gene clustering heat map, (D) The histogram of GO enrichment, (E) The histogram of KEGG enrichment in *V. parahaemolyticus* cells after combined ultrasound with FeSO_4_ treatments.

To further understand the mechanisms by which US + FeSO_4_ treatments could induce ferroptosis in *V. parahaemolyticus*, we conducted a comprehensive analysis of transcriptome alterations by RNA sequencing. The distribution of differential expressed genes between control group and treated group were visualized by a volcano plot and heat maps. As shown in Fig. 7A-C, US+FeSO_4_ treatments resulted in obvious global changes compared with the control group. A total of 1846 DEGs were screened, including 912 up-regulated genes and 934 down-regulated genes. GO analysis showed that most DEGs were mainly clustered in oxidoreductase activity, type II protein secretion system complex, Gram-negative-bacterium-type cell outer membrane assembly, rRNA binding, iron ion binding, protein folding and other metabolism pathways (Fig. 7D). KEGG analysis revealed that in addition to the special ferroptosis pathway was significantly influenced, these genes also concentrated in TCA cycle, carbon metabolism, fatty acid metabolism, biofilm formation, RNA degradation, two-component system, oxidative phosphorylation, ribosome (Fig. 7E). These pathways were proven to play vital roles in oxidative stress response, lipid peroxidation, DNA damage, iron metabolism and some other important biological processes related to cell death.

### 3.8 The effects of US combined with FeSO_4_ on the quality of salmon

**Fig. 8:**
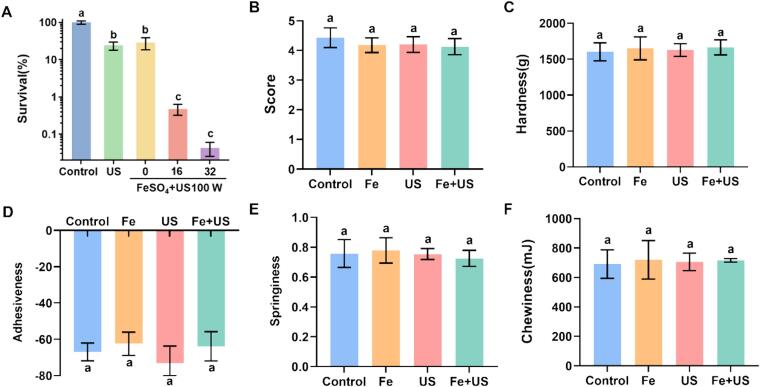
Effect of ultrasound and FeSO_4_ treatments on the quality of salmon. (A) The survival of *V. parahaemolyticus* on salmon after ultrasound and FeSO_4_ treatments. (B) Sensory analysis of salmon after ultrasound and FeSO_4_ treatments. (C) Hardness of salmon. (D) Adhesiveness of salmon. (E) Springiness of salmon. (F) Chewiness of salmon. Different letters above the bars indicated that the mean values of three replicates were significantly different among different treatments (*p* < 0.05).

To simulate the potential applications in the food industry, we evaluated the validity of US+FeSO_4_ treatment to kill *V. parahaemolyticus* on salmon. The result showed that the survival in treated group had a significant reduction, almost 99.9 % *V. parahaemolyticus* were killed after treated with US (100 W) + FeSO_4_ (32 μM) (Fig. 8A). Meanwhile, the effect of different treatments on the quality of salmon were evaluated. As shown in Table 1, there were no significant differences in the values of L*, a*, and b* between different groups, indicating that US+FeSO_4_ had almost no effect on the color of salmon. The sensory analysis was carried out by 10 food experts. Compared with the control group, there was little change in the sensory properties of salmon in treated groups. In addition, the different treatments led no obvious changes in hardness, adhesiveness, springiness, chewiness of salmon. Therefore, our reports provided a potential basis for applying this method to control *V. parahaemolyticus* in raw-fish products.

**Table 1:** Effect of different treatments on the color of salmon.

Treatment	L*	a*	b*
Control	51.24 ± 0.55^a^	14.02 ± 0.25^a^	17.27 ± 0.47^a^
US	50.46 ± 0.36^a^	13.54 ± 0.14^a^	17.48 ± 0.97^a^
FeSO_4_	50.77 ± 0.86^a^	13.15 ± 0.35^a^	17.83 ± 0.20^a^
US+FeSO_4_	50.50 ± 0.37^a^	13.70 ± 0.46^a^	16.71 ± 0.08^a^

Different small letters in the same column mean significantly different (P<0.05).

## 4 Discussion

Ultrasound has been widely used in the food industry due to its potential efficacy in deactivating microorganisms present in various food products [29]. However, some studies found that the mechanical effects by ultrasound without damaging the quality of the product are insufficient for the antimicrobial effect [14], [30]. It can be utilized in combination with other antimicrobial methods to constitute a synergistic effect [31], [32]. Iron as the essential micronutrient has been found to efficiently induce ferroptosis-like cell death in food-borne pathogens, such as *Staphylococcus aureus* [33], *Escherichia coli* [34], *V. parahaemolyticus* [13], *Pseudomonas aeruginosa* [35] and *Aspergillus flavus* [22]. To our knowledge, there are no reports on the potential effect of combining ultrasound and FeSO_4_.

In the present study, FeSO_4_ (2–16 μM, 15 min) treatments and US (60–100 W, 15 min) treatments alone showed limited bactericidal activities on *V. parahaemolyticus*. Previous studies reported that FeSO_4_ exhibited remarkable antibacterial activity against *V. parahaemolyticus* after 180 min of treatment [13]. Our findings are in accord with previous studies, suggesting that the bactericidal activity of FeSO_4_ was related to the treatment time. Meanwhile, in the study of Yang et al. [36], the number of *S. Typhimurium* was reduced by only 0.41–1.81 log CFU/mL after 2–8 min of US (115–253 W/cm^2^) treatment. Li et al. [37] reported that only 0.47––0.52 log CFU/mL bacteria were reduced after 8 min of US (60 W/cm^2^) treatment. Liu et al. [38] found that ultrasonic treatment had a significant lethal effect on *V. parahaemolyticus* with the increase of power density, almost 45 % of *V. parahaemolyticus* cells were killed after 20 min of treatment at 7.5 W/mL power density. In this study, we reported an apparent synergistic effect of US+FeSO_4_ treatment, and the sterilization rate of *V. parahaemolyticus* was more than 99.9 % treated by US (100 W, 15 min) combined with 8 μM FeSO_4_. Therefore, our results indicated that the combination of US and FeSO_4_ might be a novel bactericidal strategy, which not only can achieve significant sterilization effects, but also reduce the treatment time and the power of ultrasound. Our results would provide insights into the synergistic application of ultrasound and FeSO_4_ to control other food-borne pathogens contamination in food industry.

Ferroptosis is a form of programmed cell death driven by iron-dependent lipid peroxidation culminating in membrane rupture, which differs from apoptosis, necrosis, and autophagy [8], [39]. Our study uncovered that US+FeSO_4_ treatment had a stronger bactericidal effect against *V. parahaemolyticus*, accompanied by the emergence of ferroptosis hallmarks, including iron-dependent, ROS burst and lipid accumulation. And the specifical ferroptosis inhibitor Lip-1 could obviously alleviate cell death induced by US+FeSO_4_, confirming the occurrence of ferroptosis. Notably, although previous studies have demonstrated that FeSO_4_ could induce ferroptosis-like death in *V. parahaemolyticus*, the eruption of ROS and lipid peroxidation accumulation were not observed [13]. A possible explanation for this might be that the introduction of ultrasound influenced glutathione peroxidase 4 (GPX4) pathway, a ferroptosis inhibition by reducing phospholipid hydroperoxide and hence repressing lipoxygenase-mediated lipid peroxidation[40], [41]. Moreover, recent study has indicated that ultrasonic cavitation could induce an apoptosis-like death process in *V. parahaemolyticus* entering via SOS response, with a significant increase in ROS level post ultrasound treatment [38]. GPX4, functioning as an antioxidant enzyme, plays a crucial role in protecting cells from oxidative stress [42]. It was reported that GPX4 overexpression could inhibit ROS-induced cell death [43]. These findings further supported the idea above. What’s more, the addition of FeSO_4_ completely altered the type of cell death induced by ultrasound. Collectively, these data highlighted that the combination of ultrasound and FeSO_4_ promoted a novel form of death-ferroptosis.

How does the combination of ultrasound and FeSO_4_ trigger ferroptosis in *V. parahaemolyticus*? We hypothesized that this might be attributed to the mechanical effect of ultrasound. The intense liquid shear and shock waves generated by ultrasonic waves can enhance the permeability of cell membranes and form transient pores [44], which may promote easier entry of Fe^2+^ into bacterial cells. Fe^2+^ eruption is critical event in ferroptosis. Supporting this hypothesis, upon labeling with the FeRhoNoxTM-1 probe, we observed a remarkable increase in intracellular Fe^2+^ following treatment with US+FeSO_4_. Consistent with this, Hou et al. [45] reported that ultrasound enhanced the penetration of NaClO into *Listeria Monocytogenes*. Li et al. [46] showed that the gap created by ultrasound allowed β-citronellol to enter the cell more easily. He et al. [47] found that ultrasound-induced membrane pores facilitated the entry of thyme essential oil nanoemulsion into *E. coli*. Moreover, the rupture of cavitation bubbles generates free radicals may also be responsible for the occurrence of ferroptosis [19]. The free radicals are transported to the microbial cells, causing the increase of ROS level and cellular oxidative damage. Subsequently, free radicals generated by ultrasound and Fenton reaction resulted in the lipid peroxidation accumulation and the occurrence of ferroptosis.

Meanwhile, the transcriptome analysis further elucidated the response mechanism underlying ferroptosis in *V. parahaemolyticus*. Notably, pathways directly implicated in ferroptosis, such as fatty acid metabolism, biofilm formation, RNA degradation, oxidative phosphorylation were significantly affected. Apart from that, DEGs implicated in other pathways might also indirectly contribute to the occurrence of ferroptosis, including two-component system, carbon metabolism, ribosome metabolism. Two-component system is mainly responsible for sensing the environment changes and regulating the expression of certain genes [48]. For instance, the EnvZ/OmpR system regulates pore proteins in the outer membrane that facilitate the entry of hydrophilic substances, potentially including Fe^2^ [49]. Decreases in carbon metabolism have been observed in bacteria undergoing programmed cell death [50]. Ribosome metabolism plays an important role in responding challenging to environments and reducing oxidative damage [24]. These findings provide valuable insights into how this unique treatment (US+FeSO_4_) triggers ferroptosis and its underlying molecular pathways.

## 5 Conclusions

In summary, this study investigated US treatment in combination with FeSO_4_ had an excellent synergistic effect on the inactivation of *V. parahaemolyticus*. The combination treatment-induced cell death was identified as a novel form of ferroptosis, with the emergence of ferroptosis hallmarks including iron-dependent, ROS burst and lipid peroxide accumulation. The bactericidal mechanism was attributed to the mechanical effect of ultrasound, which promoted the entry of Fe^2+^ into bacterial cells and the formation of free radicals. Our findings propose a novel approach for the disinfection and sterilization of food products.

## CRediT authorship contribution statement

**Shurui Peng:** Writing – original draft, Methodology, Investigation, Data curation, Conceptualization. **Lishan Yao:** Writing – review & editing, Methodology, Formal analysis, Data curation. **Xiaolin Zhu:** Funding acquisition, Formal analysis, Data curation. **Wei Ge:** Writing – review & editing, Data curation, Conceptualization. **Jiakun Deng:** Writing – review & editing, Data curation, Conceptualization. **Hongbo Li:** Writing – review & editing, Investigation. **Dan Xu:** Writing – review & editing, Visualization. **Liangbin Hu:** Writing – review & editing, Project administration, Methodology, Conceptualization. **Haizhen Mo:** Writing – review & editing, Methodology, Investigation, Funding acquisition, Data curation, Conceptualization.

## Declaration of competing interest

The authors declare that they have no known competing financial interests or personal relationships that could have appeared to influence the work reported in this paper.
